# The Epidemiology of Ocular *Chlamydia trachomatis* Infection within Districts Persistently Endemic for Trachoma in Amhara, Ethiopia

**DOI:** 10.4269/ajtmh.23-0876

**Published:** 2024-07-02

**Authors:** Scott D. Nash, Eshetu Sata, Ambahun Chernet, Tania A. Gonzalez, Andrew W. Nute, Victoria C. Ontiveros, Demelash Gessese, Mulat Zerihun, Kimberly A. Jensen, Gizachew Yismaw, Taye Zeru, Berhanu Melak, Zebene Ayele, Fetene Mihretu, Fikre Seife, Zerihun Tadesse, E. Kelly Callahan

**Affiliations:** ^1^ The Carter Center, Atlanta, Georgia;; ^2^ The Carter Center, Addis Ababa, Ethiopia;; ^3^ Amhara Public Health Institute, Bahir Dar, Ethiopia;; ^4^ Federal Ministry of Health, Addis Ababa, Ethiopia

## Abstract

Persistent trachoma is a growing concern to trachoma control programs globally and programs serving Ethiopia specifically. Persistent trachoma is defined as a district with two or more trachoma impact surveys (TISs) at which the prevalence of trachomatous inflammation-follicular (TF) among children ages 1–9 years is ≥5%, the elimination threshold. Because the global target for trachoma elimination as a public health problem is 2030, research is needed to better characterize persistent trachoma. This study described the epidemiology of ocular *Chlamydia trachomatis* infection, the causative bacteria of trachoma, in seven contiguous districts experiencing persistent trachoma. In 2019, multistage cluster random sampling TISs were conducted in the seven districts after 10 years of interventions. All individuals ages ≥1 year were examined for trachoma clinical signs by certified graders, and conjunctival swabs were collected from children ages 1–5 years to test for *C. trachomatis* infection. The district TF prevalence ranged from 11.8% (95% CI:7.6–16.0%) to 36.1% (95% CI:27.4*–*44.3%). The range of district-level *C. trachomatis* infection prevalence was between 2.7% and 34.4%. Statistically significant spatial clustering of high*-*infection communities was observed in the study districts, and children with infection were more likely than those without to be found in households with clinical signs of trachoma and those without latrines. These seven districts appear to constitute a persistent hotspot in Amhara, where an additional 3*–*5 years or more of interventions will be required. The global program will need to strengthen and enhance intervention strategies within persistent districts if elimination by 2030 is to be achieved.

## INTRODUCTION

The global trachoma control program has observed remarkable progress in reducing trachoma across many endemic countries since the establishment of the Surgery, Antibiotics, Facial cleanliness, and Environmental improvement (SAFE) strategy. To date, 17 countries have been validated by the WHO as having eliminated trachoma as a public health problem.^1^ As of 2022, the global population living in trachoma-endemic areas was estimated at 132 million people, and 52% of these people were in Ethiopia.^1^ The Amhara region of Ethiopia has historically had the highest burden of trachoma in the country.^2^ Prior to the start of the Trachoma Control Program in Amhara, all districts were demonstrated to be highly endemic.^3^^,^^4^ Since going to scale with SAFE regionwide in 2010, significant progress has been observed, with nearly 30% of the region having reached the threshold for trachoma elimination as a public health problem as of 2020.^5^

Despite the progress observed in Amhara, a considerable number of districts in the region are experiencing persistent trachoma. Persistent trachoma was recently defined as a district under the SAFE strategy with at least two trachoma impact surveys (TISs) at which the prevalence of trachomatous inflammation-follicular (TF) among children ages 1–9 years was at or above the elimination threshold of 5%.^6^ Trachoma impact surveys are typically conducted at the district (administrative unit for healthcare delivery) level after 3–5 years of SAFE interventions. Persistent trachoma is increasingly being recognized as a threat to the global elimination of trachoma.^5^^,^^7^^–^^10^ Recent recommendations from a WHO-convened informal consultation called for increased inclusion of alternative indicators such as monitoring *Chlamydia trachomatis* infection within surveys of trachoma-persistent districts.^6^ The Amhara Trachoma Control Program has included *C. trachomatis* infection monitoring as part of routine TISs since 2011.^10^^,^^11^

To reach elimination of trachoma as a public health problem by 2030, it is urgent to have a better understanding of persistent trachoma. The aim of this study was to examine the epidemiology of ocular *C. trachomatis* infection at the district, community, and individual levels in Amhara within seven contiguous districts experiencing persistent trachoma after 10 years of SAFE interventions.

## MATERIALS AND METHODS

### Ethics statement.

Survey protocols for all time points were reviewed and approved by the Amhara Public Health Institute, Ethiopia, and by the Emory University Institutional Review Board (protocol 079–2006). The survey protocols for the 2019 surveys were reviewed by Tropical Data (www.tropicaldata.org/). Oral informed consent was received from heads of households or an adult member of a household for household interviews. Oral informed consent, assent, and parental consent were received from each survey participant for clinical examination and conjunctival swab sample collection, and the data were recorded electronically. Consent and assent were obtained according to the principles of the Declaration of Helsinki.

### Study setting and period.

The present study focused on seven adjacent districts located in two zones in southeastern Amhara: three districts in the South Wollo zone (Jamma, Kalala, and Wogide) and four districts in the North Shoa zone (Menz-Keya, Merhabete, Mida Woremo, and Moretna Jiru). In 2007, baseline surveys demonstrated that both zones were trachoma endemic, and accordingly, the scale-up of SAFE interventions in these zones occurred between 2007 and 2010.^3^ Surgery, Antibiotics, Facial cleanliness, and Environmental improvement interventions included community-wide mass drug administration (MDA) with antibiotics along with promotion of latrine construction and face washing through village and school-based health education programs.^5^ Since 2009, administratively reported MDA coverage has been greater than 80% for nearly all years for all districts, zonal-level coverage surveys from these zones found coverage to be greater than 80%, and district-level coverage surveys conducted in districts adjacent to this area, including Wogide, demonstrated that the highest coverage was among children ages 1–9 years (Supplemental Figure 1).^12^^,^^13^

After the implementation of the SAFE strategy, district-level impact surveys were conducted in 2014 and demonstrated that all seven districts had a TF prevalence greater than 30% (defined by the program as hyperendemic trachoma).^5^^,^^14^ As in the 2019 surveys detailed here, all districts had received 10 years of the SAFE strategy.^5^^,^^11^

### Survey design.

The sampling methodology for these surveys has been extensively described elsewhere.^5^^,^^14^ Survey sampling used a multistage design, whereby in the first stage of sampling, 30 communities (locally known as *gotts*) were chosen by using a probability proportional to the estimated size approach from community lists provided by the health centers in the respective districts.^15^ In the second stage of sampling, one segment of approximately 30 households was randomly chosen in each community. In each selected segment, all households were surveyed, and all individuals ages 1 year or older were enumerated and examined for clinical signs of trachoma. Surveys conducted in 2014 used the same two-stage selection methodology as the 2019 methods.^14^

The 2019 surveys also aimed to determine the district-level prevalence of *C. trachomatis* infection among children ages 1–5 years. Based on previous infection data from the region, a 6% infection prevalence was assumed with ±4 precision, a design effect of 3.0, and a 20% nonresponse rate; thus, 486 children were targeted per district.^10^ Therefore, conjunctival swab samples were collected from all consented and examined children in this age range residing in all 30 surveyed communities across each of the seven districts. During the 2014 survey, approximately half of the survey communities were randomly selected for swabbing, as the aim was to estimate infection at the zonal level (administrative unit that contains approximately 15 districts).^10^

### Trachoma grading training.

Integrated eye care workers participated in week-long standardized training courses before each survey round to be certified to grade trachoma based on the WHO’s simplified grading scheme.^16^ Before participating in the surveys, each trainee was required to pass both classroom and field reliability examinations.^5^ To participate in surveys, during the training, trainees had to achieve a κ greater than 0.7 for the clinical sign TF compared with a single “grader trainer” who had previously been certified by a Tropical Data master trainer.

### Data collection.

Data were electronically collected using Open Data Kit Applications on the Tropical Data application loaded on Android phones. A household-level questionnaire was used to collect water, sanitation, and hygiene (WASH) indicators. These indicators included distance to water (defined as the time it takes to retrieve water for any use and return home), the presence of a clean face (determined by the absence of both nasal and ocular discharge), and improved water source and latrine presence (based on WHO/UNICEF Joint Monitoring Program definitions for unimproved and improved water sources and sanitation facilities).^17^ Trachoma graders examined all eligible individuals for clinical signs of trachoma based on the WHO’s simplified system using ×2.5 magnification loupes and adequate light. Cases identified as active trachoma (TF and/or trachomatous inflammation-intense [TI]) were offered treatment with 1% tetracycline eye ointment to be used twice daily for 6 weeks, and cases identified as trachomatous trichiasis were referred to the nearest health post for surgical services.

### Conjunctival swab collection and laboratory procedures.

Conjunctival swabs were collected by graders trained on collecting swabs in a sterile manner using a new pair of gloves to swab each child.^11^ Gloved graders swabbed the upper left tarsal conjunctiva three times, rotating a polyester-tipped swab (Thermo Fisher Scientific, Waltham, MA) at 120 degrees along the swab axis after each swabbing of the conjunctiva. The swab samples were then labeled, placed dry into a labeled 2.0-mL tube, and put inside a cooler bag with ice packs. While in the field, the samples were stored in vaccine coolers and then transferred to −20°C freezers in a laboratory until testing. A negative field-control “air-swab” was collected in every cluster by twirling the swab (swabbing the air) in front of the conjunctivae of a randomly selected child.^11^

All laboratory testing was performed at the Trachoma Molecular Laboratory at the Amhara Public Health Institute in Bahir Dar, Ethiopia. Between October 2019 and March 2020 conjunctival swabs from each district were randomized and rehydrated with 1.0 mL of molecular grade water, and then 100 µL from five swab specimens were combined into each pool. Pools were processed with the RealTime (Abbott Molecular Inc., Des Plaines, IL) polymerase chain reaction assay on the Abbott m2000 system to estimate the district-level prevalence of *C. trachomatis* infection. One district had a high pooled prevalence (80% or above); thus per protocol, samples were re-pooled into pools of three to increase accuracy.^10^^,^^18^ Laboratory technicians were masked to the clinical outcomes of individuals included in the survey, their district of residence, and whether a sample was a survey or control swab. Control samples were tested in the same exact manner as survey swabs. Laboratory and quality control procedures have been described previously.^10^^,^^11^^,^^19^^,^^20^

Samples from positive pools were assayed individually from three of the seven districts between September and October 2020 with a focus on those districts with the highest district-level infection prevalence. This was possible for only three districts owing to resource constraints. Prior to individual testing, a standard set of elementary body (EB) titrations were assayed on the m2000 to create a calibration curve, as described previously, to obtain a quantitative measure of *C. trachomatis* load.^19^

## STATISTICAL ANALYSES

District-level TF prevalence estimates were provided by Tropical Data and were calculated by taking the median age–adjusted (ages 1–9 years) cluster prevalence estimates from within each district.^15^ Age-adjustment used 1-year age bands from the 2007 Ethiopian National Census population. Confidence intervals were calculated using bootstrapping methods.^15^ Prevalence estimates and CIs for TI, clean face, and other WASH variables were estimated in a manner similar to TF. District *C. trachomatis* prevalence was estimated from the pooled prevalence as the number of positive individuals most likely to have resulted in observed pooled results.^11^^,^^18^ Only point estimates are reported, as this estimation procedure does not allow for the calculation of CIs around the point estimate.^18^ The chlamydial load variable was created through the log-transformation of *C. trachomatis* EBs to reduce the heavy right skew.^21^ Community chlamydial load index took the mean of the transformed individual-level loads among positive individuals in each survey community. Pearson correlation coefficients were used to investigate the correlation between community-level infection prevalence and community chlamydial load index.

For spatial analysis, communities were separated based on the presence of *C. trachomatis* infection, and kernel density estimation for each was performed using Diggle optimal bandwidth in R.^22^ After establishing spatial autocorrelation using Global Moran’s I with 1,000 Monte Carlo simulations, empirical semivariogram was calculated to quantify the spatial variation of *C. trachomatis* infection prevalence and chlamydial load index at the community level. The range of the semivariogram represents the distance at which *C. trachomatis* prevalence stops showing spatial autocorrelation. Local indicators of spatial association (LISA) were examined with Local Moran’s I in ArcGIS Pro. Local Moran’s I is a statistic used to identify patterns of spatial autocorrelation. High-high and low-low indicate clusters of communities with similar values (either high or low), whereas high-low and low-high highlight spatial outliers where communities with high or low prevalence values are surrounded by their opposite. Zone of indifference was used to conceptualize the spatial relationship, which combined fixed- and inverse-distance–weighted methods, and Euclidean threshold distance was defined based on semivariogram. To reduce the likelihood of type I errors due to the multiple hypothesis testing inherent in the analysis (e.g., in identifying significant hotspots, cold spots, or spatial outliers), false discovery rate correction was used during the analysis at 499 permutations.

Generalized linear mixed-effects models were used to explore the correlates of *C. trachomatis* infection and log-transformed chlamydial load, controlling for the nesting of children in their respective survey communities through a random effect term for community (lme4 package in R; R Core Team, 2022).^23^ Clustering within household was not included because the large majority of households (72.5%) had only one child ages 1–5 years. The univariate models for *C. trachomatis* infection were used to identify correlates with *P*-values <0.2, which were then included in the final multivariate model. The same strategy was applied to building the univariate models and final multivariate model for log-transformed chlamydial load.

## RESULTS

In February 2019, 7,045 households were surveyed across these seven contiguous districts in Amhara (Table 1). Within these households, 6,156 children ages 1–9 years were enumerated, and 6,045 (98.2%) were examined for the signs of trachoma. Among the 3,073 children ages 1–5 years examined, 3,040 (99.0%) children were swabbed for infection testing. From the household survey, it was determined that the district prevalence of improved water source ranged from 45.2% (95% CI: 28.8–61.9%) to 75.0% (95% CI: 58.8–88.8%), whereas the district prevalence of clean face ranged from 59.4% (95% CI: 51.1–67.4%) to 70.8% (95% CI: 61.7–79.3%) (Supplemental Table 1).

**Table 1:** Survey sample size by district, Amhara, Ethiopia, 2019

Survey Unit			Enumerated		Examined			Swabbed
Zone	District	Households	All Ages	Children (1–9 years)	All Ages	Children (1–9 years)	Children (1–5 years)	Children (1–5 years)
North Shoa	Menz-Keya	1,049	4,048	938	3,579	921	441	439
North Shoa	Merhabete	1,049	3,975	884	3,436	864	455	451
North Shoa	Mida Woremo	1,048	3,830	930	3,373	915	470	454
North Shoa	Moretna Jiru	1,050	3,892	813	3,447	800	374	370
South Wollo	Jamma	1,050	3,832	875	3,381	855	438	433
South Wollo	Kalala	900	3,355	877	2,964	859	468	466
South Wollo	Wogide	899	3,359	839	2,962	831	427	427
Total	–	7,045	26,291	6,156	23,142	6,045	3,073	3,040

**Figure 1.**
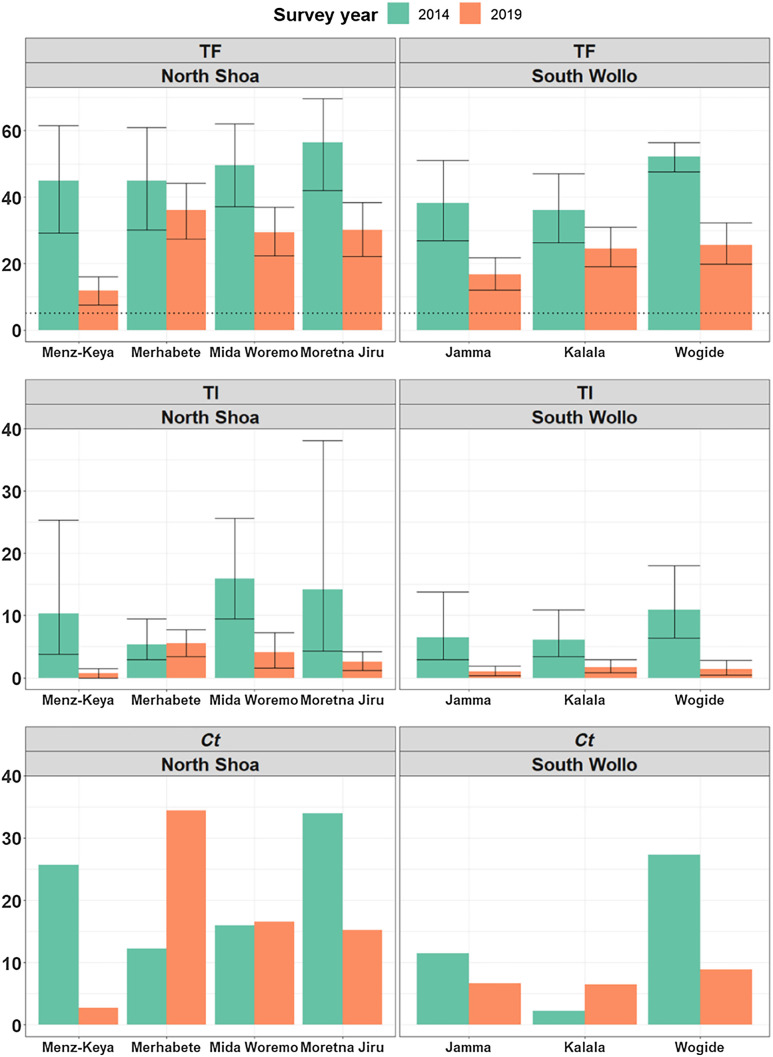
District prevalence of trachomatous inflammation-follicular (TF) and trachomatous inflammation-intense (TI) and 95% CIs among children ages 1–9 years and *Chlamydia trachomatis* (*Ct*) infection among children ages 1–5 years by zone and survey year in Amhara, Ethiopia. For infection estimates, the estimation procedures used did not allow for the calculation of CIs around the point estimate. The dotted line on the TF figures represents the 5% elimination threshold as a public health problem.

The 2019 district prevalence of TF among children ages 1–9 years ranged from 11.8% (95% CI: 7.6–16.0%) in Menz-Keya to 36.1% (95% CI: 27.4–44.2%) in Merhabete (Figure 1). Three contiguous districts in North Shoa (Merhabete, Moretna Jiru, and Mida Woremo) were close to or above the 30% hyperendemic TF threshold. District-level TF point estimates were lower in 2019 than in 2014 in all seven districts, although CIs for estimates overlapped for three of those districts. The TI prevalence among children ages 1–9 years ranged from 0.6% (95% CI: 0.01–1.4%) in Menz-Keya to 5.8% (95% CI: 3.6–7.9%) in Merhabete. Across all seven districts, the age-specific TF and TI prevalences among children were lower at all ages in 2019 compared with 2014 (Supplemental Figure 2). The 2019 district-level prevalence of *C. trachomatis* infection among children ages 1–5 years in South Wollo ranged from 6.4% in Kalala to 8.9% in Wogide, and in North Shoa the prevalence was 2.7% in Menz-Keya, 15.2% in Moretna Jiru, 16.5% in Mida Woremo, and 34.4% in Merhabete. Point estimates for *C. trachomatis* infection were lower in the second survey for four of the seven districts. None of the 210 negative control air swabs tested positive for *C. trachomatis.*

**Figure 2.**
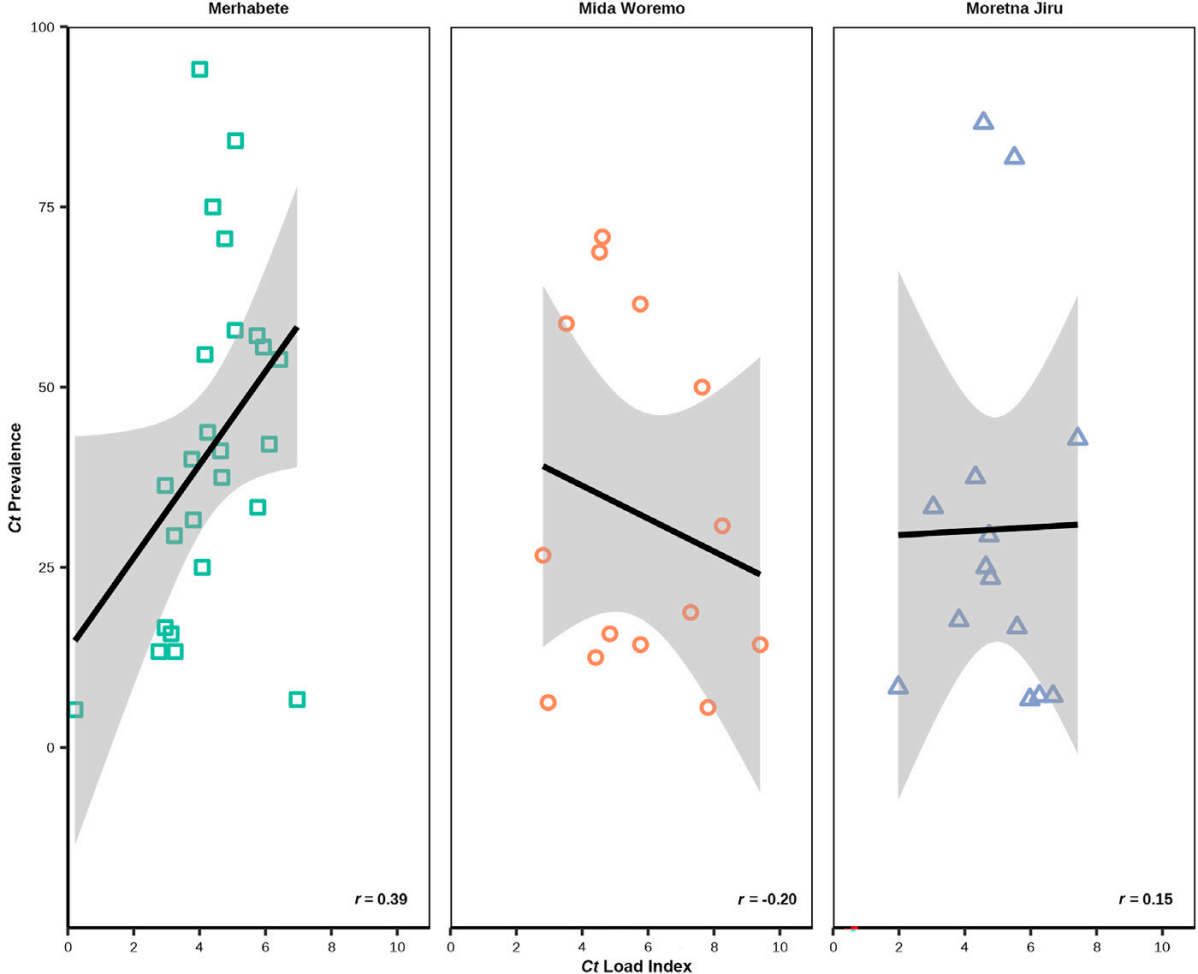
The correlation between community-level *Chlamydia trachomatis* (*Ct*) infection prevalence and community chlamydial load index among clusters with infection by survey district, Amhara, Ethiopia, 2019. The shaded area represents the 95% confidence band. *r* = correlation coefficient.

Within the hyperendemic districts of Merhabete, Mida Woremo, and Moretna Jiru, a TF prevalence of 30% or greater was found in nearly half of the surveyed communities (44/90; 48.9%). No statistically significant spatial community-level clustering was observed using Global Moran’s I or Local Moran’s I for TF prevalence (Supplemental Figure 3). Among these 90 communities, infection was detected in 54/90 (60.0%) communities; the community-specific prevalence reached as high as 94.1% in Merhabete, and the community chlamydial load index ranged from −0.06 to 9.40. The correlation between community-level *C. trachomatis* infection and community chlamydial load index was moderate (*r* = 0.39, *P* = 0.05) within communities in Merhabete but low in Mida Woremo (*r* = −0.20, *P* = 0.50) and Moretna Jiru (*r* = 0.15, *P* = 0.50) (Figure 2).

**Figure 3.**
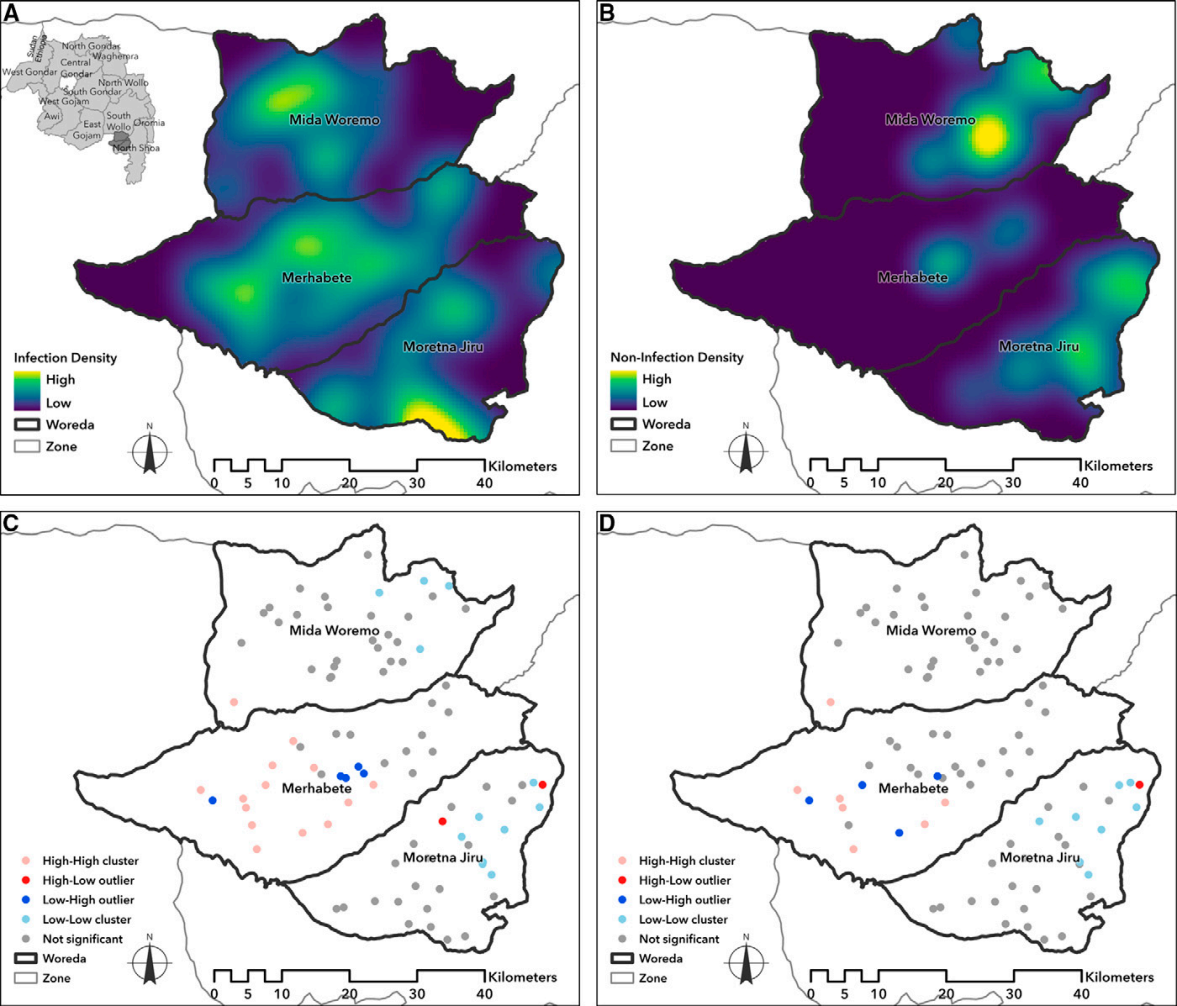
Maps of spatial density and clustering of infection outcomes among three survey districts using kernel estimation* of (**A**) infection density and (**B**) non-infection density and Local Moran’s I statistic^†^ for (**C**) infection prevalence and (**D**) community chlamydial load index, Amhara, Ethiopia, 2019. ^*^Spatial patterns between infection density and non-infection density may overlap owing to the spatial methods used to interpolate each condition. ^†^High-High indicates clusters of high-prevalence communities near high-prevalence communities, and Low-Low indicates clusters of low-prevalence communities near other low-prevalence communities. High-Low and Low-High highlight spatial outliers where high and low communities were surrounded by their opposite.

Plots of kernel density estimates based on optimized bandwidths of 3,242 m for communities with presence of infection and 2,116 m for communities without infection illustrate the spatial intensity of *C. trachomatis* infections (Figure 3). Monte-Carlo simulations for Global Moran’s I statistic indicated a positive value of 0.13 (*P* = 0.001) for infection prevalence and 0.11 (*P* = 0.001) for community chlamydial load index, indicating spatial clustering of similar values within the three-district study area. Further, LISA analysis using Local Moran’s I (with a range of influence determined by semivariogram to be 16,000 m) revealed statistically significant low-low infection prevalence clusters in the eastern portions of Moretna Jiru and Mida Woremo and significant high-high infection prevalence clusters identified in the western portion of Merhabete. Local indicators of spatial association analysis of community chlamydial load index also demonstrated similar statistically significant high-high and low-low clusters, in addition to low-high clusters in western Merhabete.

**Figure 4.**
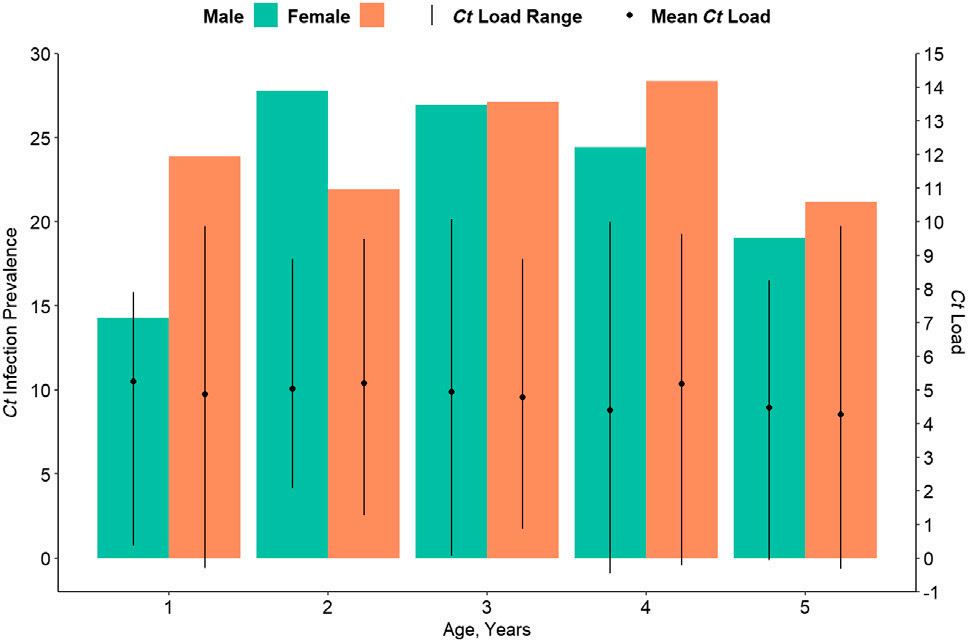
*Chlamydia trachomatis* (*Ct*) infection prevalence (left axis) and mean and range of log-transformed chlamydial load among those positive for infection (right axis) by age and sex: Merhabete, Mida Woremo, and Moretna Jiru districts, Amhara, Ethiopia, 2019.

Individual infection testing was conducted for the three districts with the highest infection prevalence. Within these districts, a total of 1,275 children ages 1–5 years were swabbed. The prevalence of *C. trachomatis* differed somewhat between females (22.3%) and males (24.4%) (*P* = 0.04) but not with age (*P* = 0.113) and was highest among children ages 3 years at 27.0% (95% CI: 21.9–32.9%) (Figure 4). Among the 298 children ages 1–5 years with detected infection, the median *C. trachomatis* load was 139.4 EBs (interquartile range = 23.9–604.1 EBs). The EB distribution was skewed in this population with maximum value of 23,696 EBs (Supplemental Figure 4). After log-transformation, the mean log-transformed chlamydial load did not differ by age (*P* = 0.09) or sex (*P* = 0.70) among this age range.

In univariate mixed-effects models controlling for community-level clustering, significant correlates of *C. trachomatis* infection among children ages 1 to 5 years were clean face, presence of latrine, presence of TF in the household, and presence of TI in the household (Table 2). In the multivariable model that included all correlates, only presence of latrine (odds ratio [OR]: 0.5; 95% CI: 0.3–0.9), presence of TF (OR: 12.3; 95% CI: 7.2–21.0), and presence of TI (OR: 3.6; 95% CI: 2.2–6.0) remained statistically significantly associated with *C. trachomatis* infection. In the second multivariate mixed-effects model among infected children, only the presence of an improved household water source was statistically significantly associated (β: −0.9; 95% CI: −1.6 to −0.2) with log-transformed chlamydial load.

**Table 2:** Correlates* of *Chlamydia trachomatis* infection and increased log-transformed chlamydial load among children ages 1–5 years: Merhabete, Mida Woremo, and Moretna Jiru districts, Amhara, Ethiopia, 2019

Survey Variables	*Ct* Infection				Log-Transformed Chlamydial Load			
	Univariate Models		Multivariable Model^†^		Univariate Models		Multivariable Model^†^	
	OR	95% CI	OR	95% CI	β	95% CI	β	95% CI
Individual Characteristics								
Age	1.0	(0.9–1.2)	–	–	0.9	(0.8–1.1)	–	–
Sex, ref = Male	1.2	(0.9–1.7)	–	–	1.1	(0.7–1.8)	–	–
Clean Face	0.7	(0.5–1.0)	1.1	(0.7–1.6)	0.7	(0.4–1.2)	–	–
Household Characteristics								
Access to Water <30 Minutes Away	0.8	(0.4–1.4)	–	–	0.5	(0.3–1.0)	–	–
Improved Water Source	1.1	(0.6–2.3)	–	–	1.5	(0.3–6.8)	−0.9	(−1.6 to −0.2)
Latrine	0.5	(0.3–0.8)	0.5	(0.3–0.9)	0.4	(0.2–0.8)	−0.6	(−1.3 to 0.0)
Presence of TF^‡^	13.0	(7.7–22.0)	12.3	(7.2–21.0)	1.7	(0.7–3.9)	–	–
Presence of TI^§^	4.1	(2.5–6.7)	3.6	(2.2–6.0)	1.7	(1.0–3.0)	0.48	(−0.1 to 1.0)

*Ct = Chlamydia trachomatis*; OR = odds ratio; ref = reference; TF = trachomatous inflammation-follicular; TI = trachomatous inflammation-intense.

* All models adjust for clustering at the community level via a random effect term.

^†^ Model containing only significant correlates (*P*-value <0.05) from univariate models.

^‡^ At least one person in household has TF.

^§^ At least one person in household has TI.

## DISCUSSION

Although reductions in TF prevalence were observed, the districts surveyed as part of this study were clearly experiencing persistent trachoma despite a decade of SAFE interventions. Ocular *C. trachomatis* infection data further supported this finding, as infection was found in all seven districts, reaching as high as 34% in Merhabete. Among the three most *C. trachomatis*–prevalent districts, surveyed communities with higher infection levels clustered in the central and western part of the study area, the presence of TF or TI in the household, and household latrine presence were correlates of infection. If the Amhara Trachoma Control Program is to achieve its goals of trachoma elimination by 2030, renewed efforts and enhanced approaches will be needed.

Ocular *C. trachomatis* infection data have been collected alongside clinical trachoma indicators since early in the Trachoma Control Program in Amhara.^4^ In 2011, the Program began incorporating conjunctival swabbing into its routine TISs to estimate the infection prevalence at zonal and regional levels. These efforts demonstrated that after approximately 5 years of interventions, the regional *C. trachomatis* infection prevalence was 5.7%, and after an additional 3–5 years the prevalence was 4.7%.^10^^,^^11^ At this second time point, the two zones that make up the current study area, South Wollo and North Shoa, demonstrated zonal *C. trachomatis* prevalences of 5.1% and 5.4%, respectively. Given the high district-level infection prevalence observed in this study, the seven contiguous districts surveyed in these zones appear to be a southern focus of persistent trachoma.^5^^,^^11^ This focus, along with the highly endemic districts in the northern zones of Wag Hemra and South Gondar, constitute major areas of concern for the Trachoma Control Program.^11^ The hyperendemic levels of TF (greater than 30%) with concomitant *C. trachomatis* infection greater than 30% in Merhabete are particularly alarming. Given that all air swabs tested negative and that the laboratory closely monitors contamination, this finding of high infection is likely robust and needs to be addressed urgently.^10^^,^^11^ Although the trachoma community has begun to define persistent trachoma as those districts that do not reach 5% TF by the second TIS, these results suggest that a new category of “hyperpersistent” may be needed.

Monitoring *C. trachomatis* infection alongside traditional indicators provided important operational information for the Trachoma Control Program in Amhara. Across the three districts with the highest district-level infection prevalence, infection was detected in approximately 60% of surveyed communities within these districts. Using spatial analysis, it was determined that although clustering of TF was not observed, communities with the highest infection prevalence were spatially clustered in the central and western parts of the study districts. Programmatic attention and operational research are especially needed within the communities found in these areas to ensure continued community support and high levels of MDA coverage. This should include the use of well-validated tools such as the data quality assessment and coverage surveys in these specific districts.^24^ At the individual level, 3-year-olds had the highest level of infection; however, considerable infection was detected across the 1–5-year age span, and infected children were more likely to be residing in households where TF and/or TI was detected. These three persistent districts would make good candidates for enhanced interventions as recommended by a recent WHO working group.^6^ Based on the results of this study, enhanced MDA strategies targeting young children and those residing in households where clinical trachoma is present would likely be an efficacious strategy. Accordingly, the Program is helping to support academic and regional partners in conducting a randomized trial of targeted enhanced antibiotic strategies in this area (clinicaltrials.gov NCT03335072).

The infection assay used at the Trachoma Molecular Laboratory in Amhara allowed for the additional quantification of the infectious load found among individuals and communities. The distribution of EB load within children in the three high-infection districts was highly skewed, with most infections being of low load. This distribution has been observed in other settings of varying endemicity and was similar to that of a previous study in Amhara that encompassed districts with both high- and low-infection prevalence.^19^^,^^25^^–^^27^ As chlamydial load was similar across the age span in this study, it is likely even children as young as 1 year are contributors to ongoing transmission in trachoma-persistent districts. Community chlamydial load index is not a common indicator in trachoma studies, although it was recently used as a randomized trial outcome in Amhara.^21^^,^^28^ Community chlamydial load index has advantages in that it can help programs identify communities with the heaviest burden of infection, and thus those most likely to maintain transmission.^28^ In this study, community chlamydial load index was only moderately associated with community-level infection prevalence, and statistically significant individual-level correlates were different for each outcome. This suggests that the epidemiology may be different within these communities, those with high-infection prevalence versus those experiencing high load burdens. Longitudinal studies or sentinel monitoring would likely be needed to understand the contributions of high-load communities to the continued persistence of trachoma within a district.

The WASH indicators collected in these surveys demonstrated that much work remains in this part of Amhara to provide adequate water and sanitation. Most households living in these districts did not have latrine access and were traveling more than 30 minutes to access water; and in three of the seven districts, less than half of the households had access to improved water sources. Within the study districts, having a household latrine was associated with lower odds of *C. trachomatis* infection. An absence of a latrine may be a generalized indicator of poor sanitation, and open feces have been demonstrated to be preferred larval medium for the trachoma-transmitting *Musca sorbens* fly.^29^ In previous work in the region, increased community coverage of latrines was associated with a lower trachoma burden.^30^ Although previous reports from Amhara have demonstrated increases in latrine coverage since the start of SAFE interventions, clearly more work is needed to provide these types of latrines recommended by the WHO.^5^^,^^31^ In another previous report from Amhara, it was determined that lower levels of water access were a correlate of trachoma hotspots regionwide.^32^ In this study, infected children within households with improved water sources were likely to have lower chlamydial loads after adjusting for household level TF. This suggests that increased water access may help limit high-load infections. Clearly, WASH improvements are needed in these districts, and in Amhara research is underway to better understand the effects of water provision on *C. trachomatis* infection within hyperendemic districts (clinicaltrials.gov NCT02754583).^21^

This study had some limitations. Direct comparison of trachoma indicators over time should be done with caution. Although the TISs used similar methodology at the two time points, TISs are designed to estimate point prevalence, not change over time. However, the result of the two surveys can be used to mark progress over time toward the 5% elimination threshold under the SAFE strategy. Fewer children per district were swabbed in the first survey than in the second survey, although they were sampled in an unbiased way.^10^ Because of this increased imprecision in the earlier estimate, it is even more difficult to compare infection prevalence across the two time points. The *C. trachomatis* infection prevalence at the second survey, measured among all 30 survey clusters per district, was nearly 3% or greater for all districts, demonstrating that transmission is likely ongoing in all seven districts after considerable intervention pressure. Monitoring large-scale programs using *C. trachomatis* infection added additional costs in the form of materials, additional training, and additional team members, as well as storage and testing costs. A cost comparison of TISs with and without conjunctival swabbing is needed and is underway in the region. Lastly, because of cost limitations, individual *C. trachomatis* data were available from the three most endemic (TF of 29.4% or greater) districts. Correlates of infection may be different within persistent districts with lower endemicity. Despite these limitations, this study used an infection assay that is highly sensitive and specific, with a continually demonstrated good quality control profile.^10^^,^^11^ Furthermore, all work was completed within the region by the Trachoma Control Program. Through the programmatic monitoring of *C. trachomatis* infection, a deeper understanding of the epidemiology of trachoma in persistent settings was possible.

This study determined that persistent districts in this part of Amhara were characterized by considerable *C. trachomatis* infection, that spatial clustering was evident, and that programs should target interventions to communities with high levels of clinical trachoma and low WASH indices to have the most effect on infection. Because 10 years of standard SAFE interventions have not reduced infection to a low level or TF to below 5%, improvements and enhancements to this strategy are needed if elimination by 2030 is to be achieved.

## Supplemental Materials

10.4269/ajtmh.23-0876 Supplemental Materials
