# Supplementary material for: The Epidemiology of Ocular Chlamydia trachomatis Infection within Districts Persistently Endemic for Trachoma in Amhara, Ethiopia
Source: Am J Trop Med Hyg. 2024 Jul 2;111(3 Suppl):105–13. doi: 10.4269/ajtmh.23-0876 (PMC11376115; doi:10.4269/ajtmh.23-0876)
Supplement: Supplemental Materials [file tpmd230876.SD1.pdf]

Supplemental Figure 1. Administrative mass drug administration (MDA) coverage by district, 2009-2019, Amhara, Ethiopia, 2019

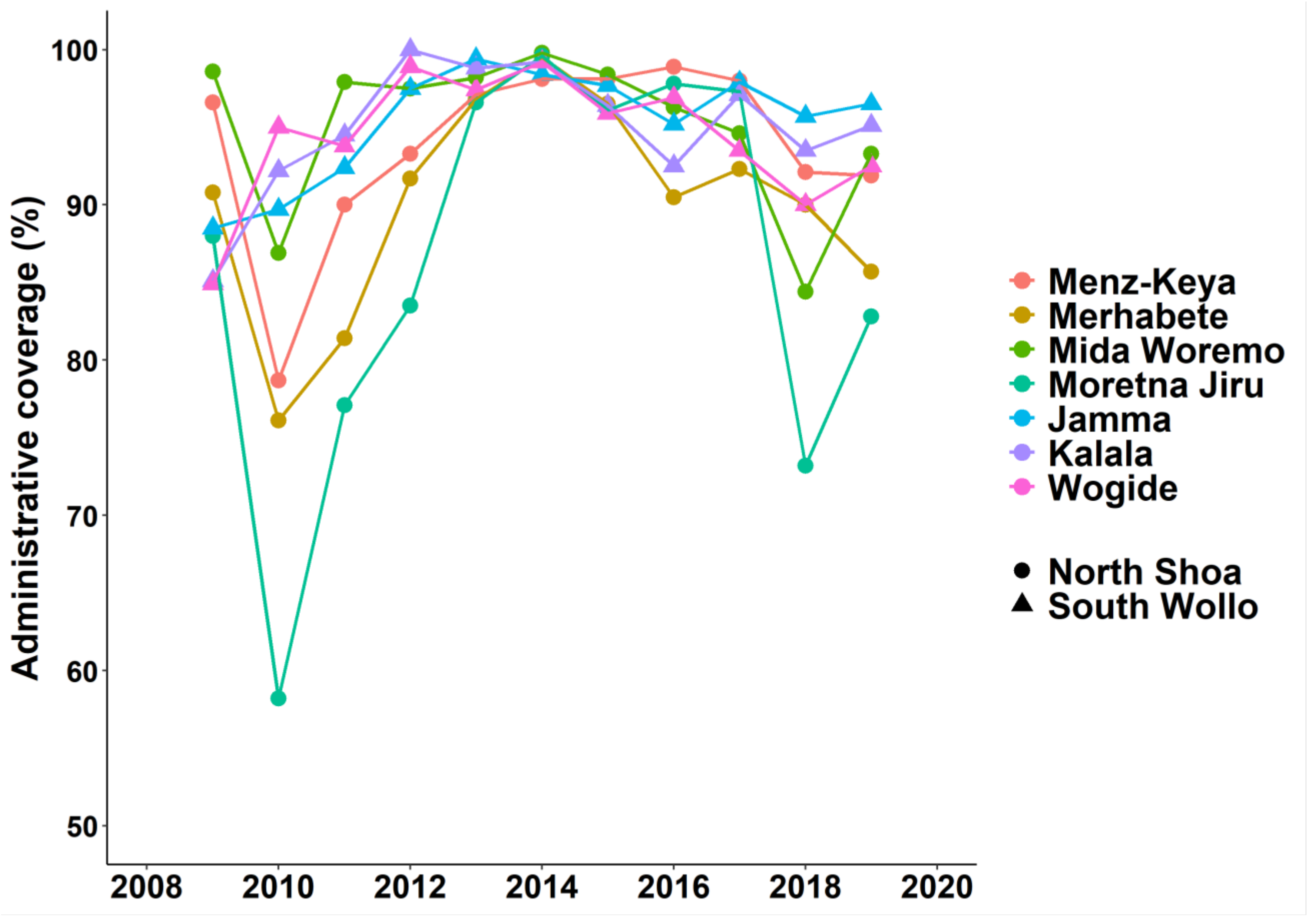


Administrative coverage defined as the total number of doses distributed divided by the targeted population.

Supplemental Figure 2. Age specific prevalence of trachomatous inflammation-follicular (TF) and trachomatous inflammation-intense (TI) among children ages 1 to 15 years across 7 districts of Amhara, Ethiopia, 2019

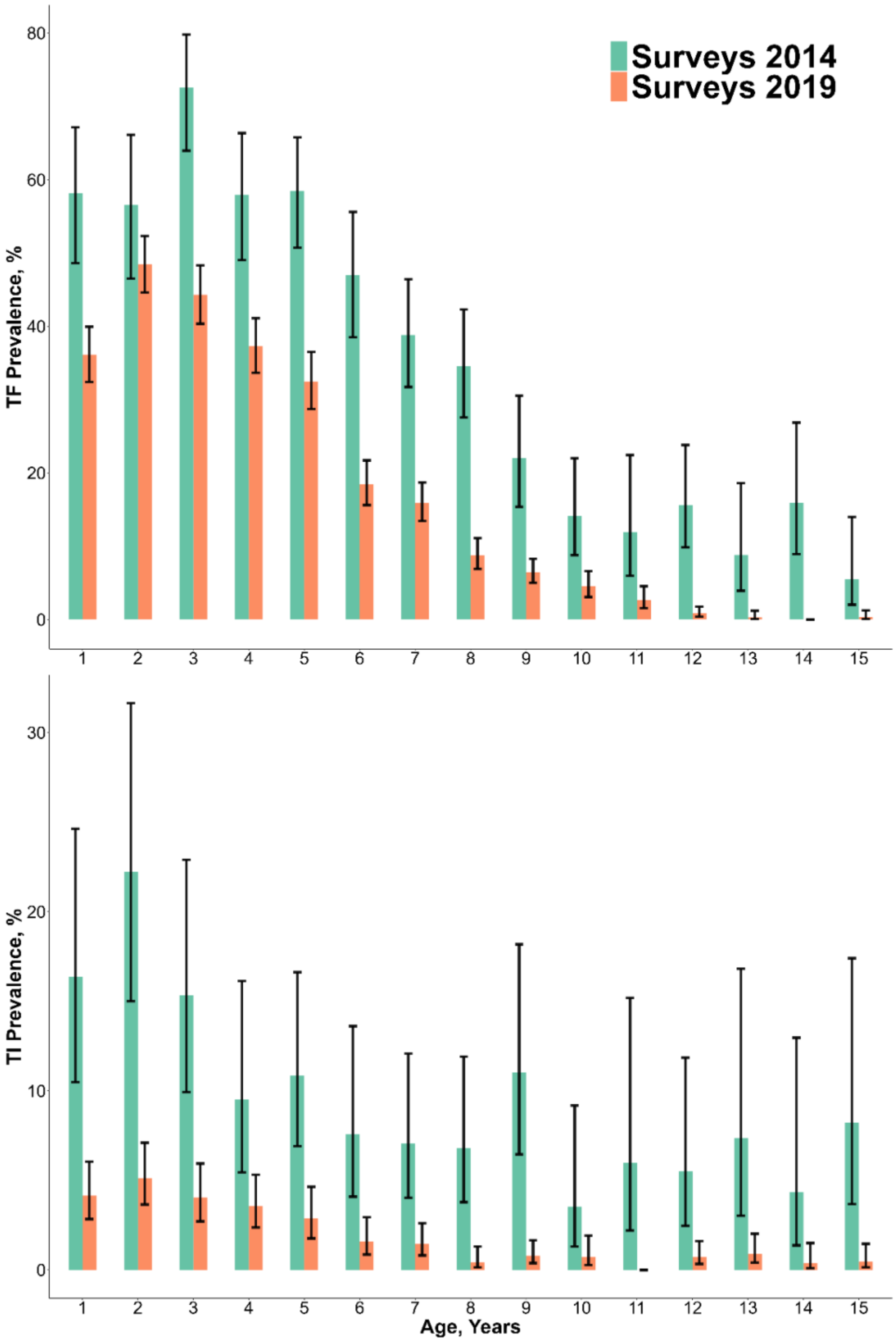

Supplemental Figure 3. Maps of spatial density and clustering among 3 survey districts using kernel estimation of a) trachomatous inflammation-follicular (TF) density and b) non-TF density, Amhara, Ethiopia, 2019

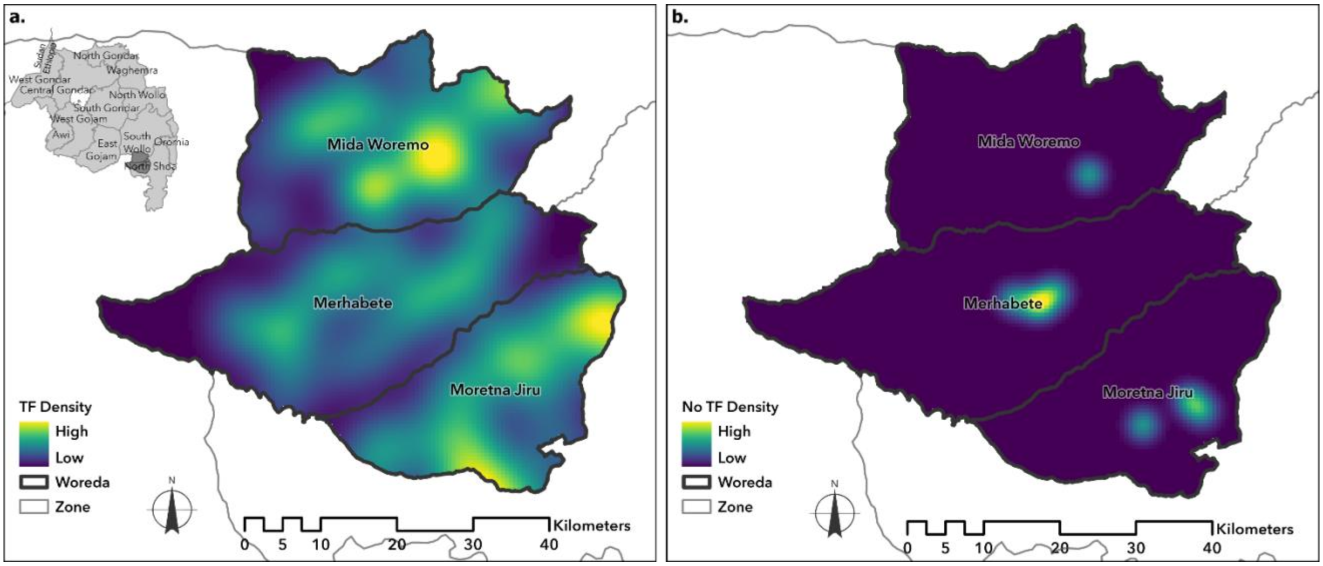

Supplemental Figure 4. Distribution of *Chlamydia trachomatis* elementary body load, Amhara, Ethiopia, 2019

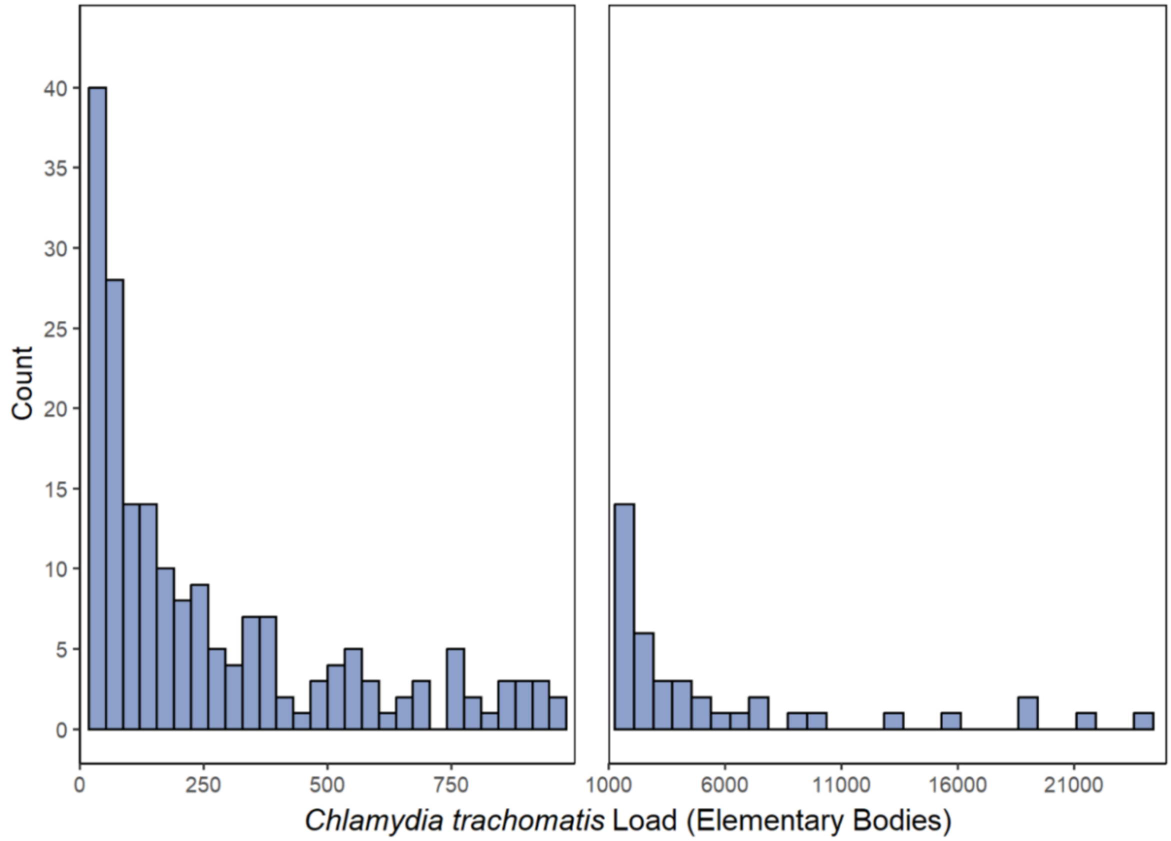

Supplemental Table 1. District prevalence of water, sanitation, and hygiene indicators, Amhara, Ethiopia, 2019

| District | Clean face, ages 1-9 years (95% CI) | Presence of latrine (95% CI) | Improved water source (95% CI) | Access to water < 30 minutes (95% CI) |
|---|---|---|---|---|
| Menz-Keya | 59.4 (51.1, 67.4) | 36.6 (27.4, 45.7) | 47.2 (31.6, 63.0) | 26.2 (15.2, 36.8) |
| Merhabete | 63.4 (54.9, 71.9) | 22.9 (14.0, 33.2) | 69.2 (56.3, 85.8) | 22.6 (11.8, 36.0) |
| Mida Woremo | 66.4 (58.4, 74.3) | 34.4 (25.0, 44.4) | 45.2 (28.8, 61.9) | 21.4 (11.7, 33.9) |
| Moretna Jiru | 65.9 (58.7, 72.7) | 40.7 (30.1, 51.6) | 57.0 (41.6, 74.5) | 23.6 (11.0, 36.2) |
| Jamma | 70.8 (61.7, 79.3) | 64.3 (54.0, 74.3) | 71.6 (57.9, 86.6) | 26.3 (14.3, 38.2) |
| Kalala | 69.6 (61.8, 77.2) | 43.8 (31.8, 56.1) | 48.3 (32.0, 65.8) | 23.1 (11.1, 35.3) |
| Wogide | 68.2 (61.3, 74.6) | 38.7 (27.3, 50.6) | 75.0 (58.8, 88.8) | 30.2 (16.1, 42.8) |
